# Appropriate Implantable Cardioverter-Defibrillator Therapy in Patients with Ventricular Arrhythmia of Unclear Cause in Secondary Prevention of Sudden Cardiac Death

**DOI:** 10.3390/jcm12134479

**Published:** 2023-07-04

**Authors:** Alwin B. P. Noordman, Michiel Rienstra, Yuri Blaauw, Bart A. Mulder, Alexander H. Maass

**Affiliations:** Department of Cardiology, Heart Center, University of Groningen, University Medical Center Groningen, 9713 GZ Groningen, The Netherlands; a.b.p.noordman@umcg.nl (A.B.P.N.); b.a.mulder@umcg.nl (B.A.M.)

**Keywords:** implantable cardioverter-defibrillator, secondary prevention, etiology, idiopathic VF

## Abstract

In this study, we sought to investigate the occurrence of appropriate implantable cardioverter-defibrillator (ICD) therapies and inappropriate shocks in secondary prevention ICD recipients with ventricular arrhythmia of unclear cause and ventricular arrhythmia in the context of underlying heart disease. In this retrospective study, consecutive patients with an ICD implanted for secondary prevention in the University Medical Center Groningen (UMCG), the Netherlands between 1 January 2012 and 31 December 2018 were included. Patients were classified as having ventricular arrhythmia of unclear cause if no clear cause was found which could explain the index ventricular arrhythmia. The primary outcome was appropriate ICD therapy. The study population consisted of 257 patients. In 220 patients, an underlying heart disease could be identified as the cause of ventricular arrhythmia, while 37 patients had an unclear cause of ventricular arrhythmia. The median age was 64 years (interquartile range (IQR) 53–72 years). Forty-five (18%) patients were women. During a median duration of follow-up of 6.2 years (IQR 4.8–7.8 years), appropriate ICD therapy occurred in 95 (37%) patients. This number was 90 (41%) in the group with a clear etiology and 5 (14%) in the group with an unclear etiology. In multivariable analysis, index ventricular arrhythmia of unclear cause was associated with fewer appropriate ICD therapies (HR 0.37 [95% CI 0.14–0.99]; *p* = 0.048), as well as an increased risk of inappropriate ICD shocks (HR 3.71 [95% CI 1.17–11.80]; *p* = 0.026). Index ventricular arrhythmia of unclear cause was significantly associated with fewer appropriate ICD therapies.

## 1. Introduction

Sudden cardiac death (SCD), which is defined as an unexpected death from a cardiovascular cause, is a leading cause of death and accounts for approximately half of all deaths from cardiovascular causes [1,2,3]. Ventricular tachycardia (VT) and ventricular fibrillation (VF) are ventricular arrhythmias which can lead to SCD [4]. Several underlying conditions can predispose a patient to SCD. Ischemic heart disease, comprising both acute myocardial ischemia and a previous infarction with resulting scar tissue, is the most common etiology, being responsible for 80% of fatal arrhythmias [3,4]. Other causes are non-ischemic cardiomyopathies, electrical heart diseases and congenital heart diseases [3,4,5]. However, there is also a group of patients in whom no substrate of an associated cardiovascular condition increasing the risk of ventricular arrhythmias can be found. These are patients with unexplained ventricular arrhythmias, including both VF and VT, and idiopathic VF [6,7,8,9]. Since the latter is based on exclusion of other causes, systematic diagnostic testing is of great importance [9].

Besides antiarrhythmic drugs and VT ablation, the main strategy to prevent SCD is the implantation of an implantable cardioverter-defibrillator (ICD), which detects ventricular arrhythmias and responds by delivering either a shock or antitachycardia pacing (ATP) [10]. Compared to amiodarone, ICDs reduce the number of sudden cardiac deaths by 50% [11]. Current guidelines recommend ICD implantation for secondary prevention in patients with documented VF or hemodynamically unstable VT without reversible causes [12].

It is known that the risk of SCD is highest in those who had a prior SCD, although the risk of appropriate ICD therapy varies widely [13,14,15,16,17,18,19]. It may be that this variation can, in part, be explained by the presence or absence of a clear underlying cause. Studies investigating differences in rates of appropriate ICD therapy between secondary prevention ICD recipients with a known etiology and those with idiopathic VF are lacking.

In this study, we sought to investigate the occurrence of appropriate ICD therapies and inappropriate shocks in secondary prevention ICD recipients with ventricular arrhythmia of unclear cause and ventricular arrhythmia with a known etiology.

## 2. Materials and Methods

### 2.1. Patient Population

**Figure 1:**
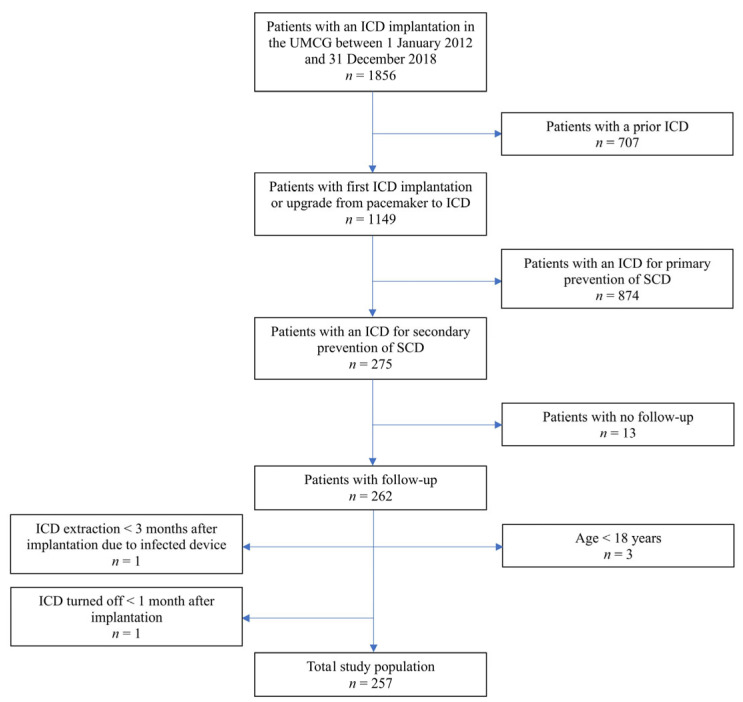
Flow diagram displaying the procedure for patient selection applied in this study. ICD, implantable cardioverter-defibrillator; SCD, sudden cardiac death; UMCG, University Medical Center Groningen.

The study population of the single-center retrospective observational study consisted of 257 consecutive patients who received their first ICD for secondary prevention of SCD in the University Medical Center Groningen (UMCG) between 1 January 2012 and 31 December 2018. The indication for ICD was discussed during multidisciplinary team meetings and based on the ESC guidelines [12]. Patients were included if all of the following inclusion criteria were met: a de novo ICD implantation or upgrade from pacemaker to ICD in the aforementioned period, documented VF or sustained VT, and age ≥ 18 years. Patients were excluded from further analysis if they had a prior ICD, if their ICD was implanted for primary prevention of SCD, if follow-up data were not available, and if age < 18 years. Patients were also excluded if their ICD was extracted within 3 months after implantation due to the device being infected or if their ICD was turned off within 1 month after implantation (Figure 1).

A waiver was obtained from the Medical Ethical Committee of the UMCG (METc 2023/141), indicating that this study does not fall under the scope of the Medical Research Involving Human Subjects Act.

### 2.2. Data Collection

Patient characteristics and clinical information at ICD implantation were collected. Follow-up data were obtained from ICD recordings and included appropriate shock and ATP, inappropriate shocks, the time until their first occurrence and the total duration of follow-up. Appropriate ICD therapy was defined as a shock or ATP given for VF or VT. Shocks or ATPs triggered by atrial fibrillation, regular supraventricular tachycardia, T-wave oversensing, or noise were registered as inappropriate. All therapies were assessed for appropriateness by two independent observers based on electrogram recordings, retrospectively or at the time of occurrence of therapy. Data were also collected from electronic medical records on all-cause mortality and the occurrence of ICD-related complications. Perioperative complications included pneumothorax resulting from device implantation and requiring intervention, lead dislocation, bleeding that required a corrective procedure or blood transfusion or cardiac perforation that occurred within 90 days after ICD implantation, while lead failure was noted when dislocation or another defect requiring intervention occurred 90 days or more after ICD implantation.

### 2.3. ICD Settings and Follow-Up

If the index arrhythmia was VF, ICDs were programmed to a therapy zone from 188 or 200 bpm with 30 intervals before detection, 2 burst ATPs with decreasing cycle length in the second burst followed by shocks at maximal output and a second therapy zone from 230 bpm with 30 intervals before detection, ATP during charging followed by shocks at maximal output. If the index arrhythmia was a monomorphic VT, ICDs were programmed to a therapy zone from a 20 ms longer cycle length than the index VT but not higher than 200 bpm with 30 intervals before detection, 2 burst ATPs with decreasing cycle length in the second burst followed by shocks at maximal output and a second therapy zone from 230 bpm with 30 intervals before detection and ATP during charging followed by shocks at maximal output.

In general, follow-up visits were scheduled every 6 months or, alternatively, every year. Home monitoring was used if available.

### 2.4. Covariate Definitions

Patients with ischemic heart disease, non-ischemic heart failure, which included DCM, as well as patients with HCM, restrictive cardiomyopathy, arrhythmogenic cardiomyopathy, electrical heart diseases, such as long QT syndrome and Brugada syndrome, congenital heart diseases, including tetralogy of Fallot and surgical corrections for congenital abnormalities, and sarcoidosis were classified as having a ventricular arrhythmia with a clear cause.

Ventricular arrhythmia of unclear cause was defined as the absence of a clear cause after extensive diagnostic testing (including history taking, electrocardiography, laboratory analysis, toxicology, echocardiography, telemetry or 24 h Holter monitoring, exercise testing, cardiac magnetic resonance imaging, ajmaline provocation test, coronary angiography, coronary artery CT angiography and genetic testing) which could explain the index ventricular arrhythmia. All cases were adjudicated by two independent observers. In general, no significant abnormalities were found using the diagnostic tests described, nor were they found during follow-up. If abnormalities were found, they were not considered sufficient explanation for the ventricular arrhythmia. In general, none of the patients with ventricular arrhythmia of unclear cause had a myocardial infarction and none had coronary artery disease that was deemed responsible for the ventricular arrhythmia. All patients had a LVEF > 45% on echocardiography or cardiac magnetic resonance imaging. None of the patients with ventricular arrhythmia of unclear cause had a genetic mutation with a corresponding phenotype which could explain the index ventricular arrhythmia. The group of patients with a ventricular arrhythmia of unclear cause consisted of patients in whom no potential cause could be found, who were considered to have idiopathic VF, and those with a potential etiology of which there was still uncertainty as to whether it was responsible for the index ventricular arrhythmia. The latter included the presence of (low amounts of) LGE on cardiac magnetic resonance imaging, presumed coronary artery spasm, coronary artery disease if it was deemed not responsible for the index ventricular arrhythmia, presumed mitral valve prolapse syndrome and presumed remnants of earlier myocarditis. In these patients, there was still uncertainty regarding the potential relationship between the found abnormality and the ventricular arrhythmia.

### 2.5. Clinical Outcomes

The first occurrence of appropriate ICD therapy, which includes shock and ATP, was the primary outcome of this study. Secondary outcomes were appropriate ICD shock, inappropriate ICD shock and all-cause mortality.

### 2.6. Statistical Analysis

Continuous data are reported as mean ± standard deviation in case of a normal distribution or median and interquartile range (IQR) in case of a skewed distribution. Dichotomous and categorical data are expressed as numbers and percentages. A *t*-test for independent groups, Mann–Whitney test and chi-square or Fisher’s exact test were performed to compare patients with an unclear versus a clear cause of ventricular arrhythmia with respect to the patient characteristics. Kaplan–Meier curves were constructed and log-rank tests were performed to analyze cumulative event-free survival. Graphs displaying the rate of appropriate ICD therapy and inappropriate shocks were constructed. Cox regression analyses were performed for all outcomes, both univariably and after adjustment for potential confounders. Potential confounders were selected based on theorical considerations and for the primary outcome included age, sex, body mass index, index arrhythmia, history of atrial fibrillation, prior syncope, history of non-sustained VT, history of myocardial infarction, QRS fragmentation, estimated glomerular filtration rate and left ventricular ejection fraction. The assumption of proportional hazards was assessed using Schoenfeld residuals. Multicollinearity was checked using Spearman’s and Pearson’s correlation coefficient for continuous variables and Phi and Cramer’s V coefficient for dichotomous and categorical variables, with a coefficient of 0.7 being used as cut-off. In all cases, variance inflation factor (VIF) was <4 and tolerance > 0.25. We checked for first-line interactions and found no significant interactions. Secondary analyses including only idiopathic VF were performed. To account for missing data, a sensitivity analysis was performed after multiple imputation.

A *p*-value < 0.05 was considered statistically significant. All statistical analyses were performed using SPSS, version 28.0 (SPSS Institute, Chicago, IL, USA) and Stata 17.

## 3. Results

### 3.1. Patient Characteristics

**Figure 2:**
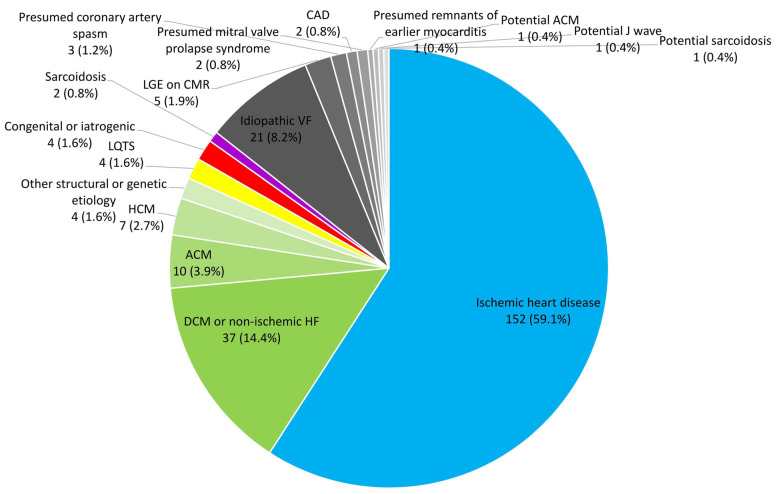
Pie chart displaying the etiologies of presenting ventricular arrhythmia for the total study population. ACM, arrhythmogenic cardiomyopathy; CAD, coronary artery disease; CMR, cardiac magnetic resonance; DCM, dilated cardiomyopathy; HCM, hypertrophic cardiomyopathy; HF, heart failure; LGE, late gadolinium enhancement; LQTS, long QT syndrome; VF, ventricular fibrillation.

The study population consisted of 257 patients. Median age was 64.1 years (IQR 52.9–71.8 years). Forty-five (17.5%) patients were women. Thirty-seven (14.4%) patients had a ventricular arrhythmia with an unclear cause. In the group with underlying cardiac disease as a substrate of ventricular arrhythmia, ischemic heart disease was the most common etiology, present in 152 (59.1%). There were more women in the group of patients with ventricular arrhythmia of unclear cause than in the group with a ventricular arrhythmia with a clear cause (11 (29.7%) vs. 34 (15.5%); *p* = 0.035). The median left ventricular ejection fraction was 45% (IQR 34–53%). VF was the presenting arrhythmia in 173 (67.3%) patients, with the remaining 84 (32.7%) having sustained VT as index ventricular arrhythmia (Table 1 and Figure 2). For a comprehensive list of patient characteristics, see Table S1.

### 3.2. Diagnostic Tests for Ventricular Arrhythmia of Unclear Cause

History taking, physical examination, electrocardiography, laboratory analysis, echocardiography and telemetry or Holter were performed in all 37 patients who had a ventricular arrhythmia of unclear cause. Coronary angiography was performed in 34 (91.9%) patients, cardiac magnetic resonance imaging in 33 (89.2%) patients, genetic testing in 19 (51.4%) patients, exercise testing in 15 (40.5%) patients, an ajmaline provocation test in 9 (24.3%) patients, toxicology in 7 (18.9%) patients and a coronary artery computed tomography angiography scan in 4 (10.8%) patients (Table 2).

### 3.3. Clinical Outcomes

The median duration of follow-up was 6.20 years (IQR 4.85–7.76 years) and was not significantly different between patients with ventricular arrhythmia of unclear cause (7.05 years (5.20–8.20 years)) and patients with ventricular arrhythmia of clear cause (5.97 years (IQR 4.77–7.71 years)) (*p* = 0.179). Appropriate ICD therapy (i.e., shock and ATP) occurred in 95 (37.0%) patients. In the group of ventricular arrhythmia with an unclear cause, the number of patients who experienced appropriate therapy was 5 (13.5%), compared with 90 (40.9%) for the group of patients with a clear cause of ventricular arrhythmia. In the idiopathic VF group, which was a subset of the group with a ventricular arrhythmia of unclear cause, 3 (14.3%) patients received appropriate device therapy. Appropriate shocks occurred in 72 (28.0%) patients. Inappropriate shocks occurred in 17 (6.6%) patients. In the group of ventricular arrhythmia with an unclear cause, this number was 5 (13.5%) patients, compared with 12 (5.5%) patients in the group with a clear cause of ventricular arrhythmia. In the idiopathic VF group, 2 (9.5%) patients experienced inappropriate shocks. 59 (23.0%) patients died during follow-up and 20 (7.8%) patients experienced device-related complications, most commonly lead failure (12 (4.7%) patients) and perioperative complications (8 (3.1%) patients) (Table 3).

**Figure 3:**
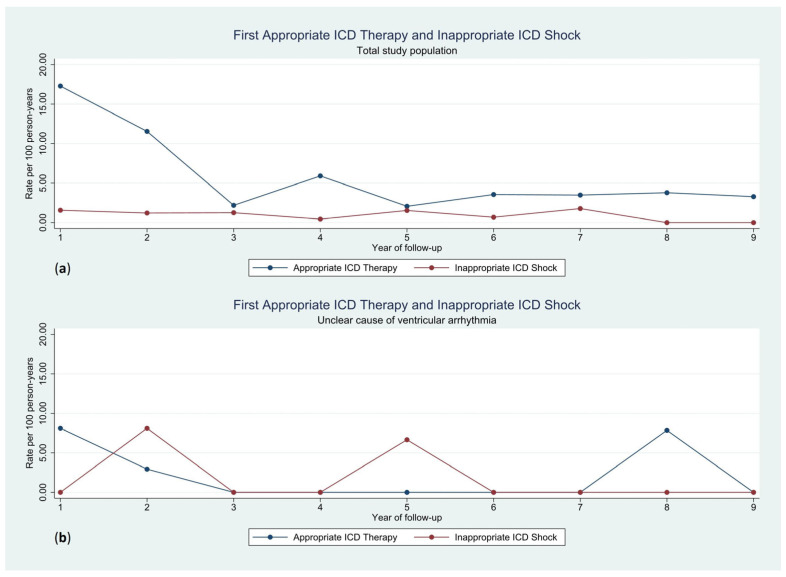
Rate of first appropriate ICD therapy and inappropriate ICD shock per 100 person-years for the total study population (**a**) and the group with an unclear cause of ventricular arrhythmia (**b**). ICD, implantable cardioverter-defibrillator.

**Figure 4:**
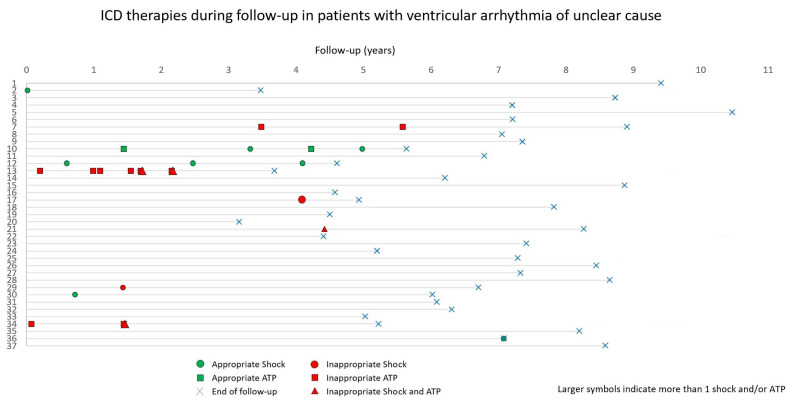
The occurrence of ICD therapies in patients with an unclear cause of ventricular arrhythmia. ATP, antitachycardia pacing; ICD, implantable cardioverter-defibrillator.

The rate of first appropriate therapies was highest during the first two years of follow-up, with a lower rate thereafter. No such temporal difference was observed for the rate of first inappropriate shocks (Figure 3). The occurrence of ICD therapies in patients with an unclear cause of ventricular arrhythmia is displayed in Figure 4.

### 3.4. Ventricular Arrhythmia of Unclear Cause

**Figure 5:**
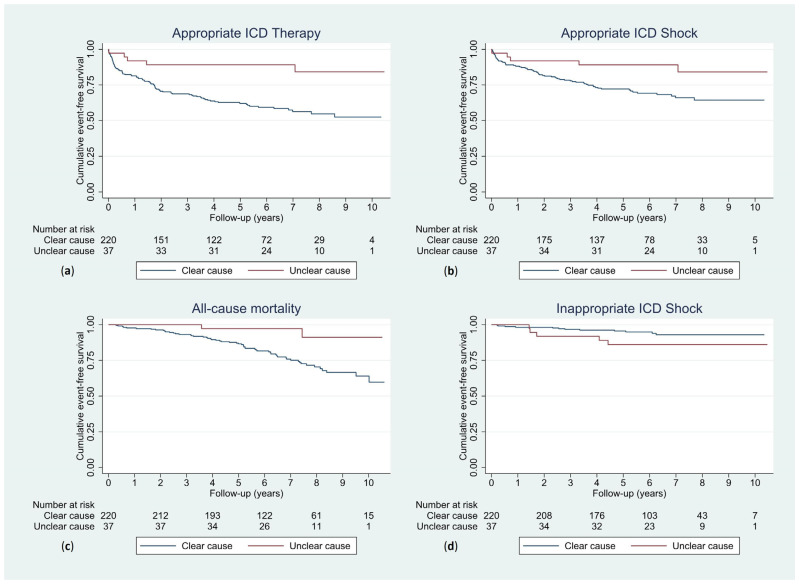
Kaplan–Meier curves displaying cumulative event-free survival for patients with a clear and unclear cause of ventricular arrhythmia with appropriate ICD therapy (log-rank test *p* = 0.002) (**a**), appropriate ICD shock (log-rank test *p* = 0.027) (**b**), all-cause mortality (log-rank test *p* = 0.010) (**c**) and inappropriate ICD shock (log-rank test *p* = 0.095) (**d**) as outcomes. ICD, implantable cardioverter-defibrillator.

Patients with an unclear cause of ventricular arrhythmia had significantly higher cumulative event-free survival rates for the outcome of appropriate ICD therapy than those with a clear cause of ventricular arrhythmia (log-rank test *p* = 0.002). For the outcome of inappropriate ICD shock, cumulative event-free survival did not differ significantly between the two groups (log-rank test *p* = 0.095), but there was a trend towards more inappropriate shocks in the unclear cause group (Figure 5).

After adjusting for potential confounders, index ventricular arrhythmia of unclear cause was associated with a lower rate of appropriate ICD therapy (univariable HR 0.26 [95% CI 0.11–0.65]; *p* = 0.004) (multivariable HR 0.37 [95% CI 0.14–0.99]; *p* = 0.048) and a higher rate of inappropriate ICD shock (univariable HR 2.37 [95% CI 0.83–6.73]; *p* = 0.105) (multivariable HR 3.71 [95% CI 1.17–11.80]; *p* = 0.026) (Table 4). In multivariable analyses, no significant associations were found for the outcomes of appropriate ICD shock and all-cause mortality (Table 4). In the sensitivity analysis with the multiply imputed datasets, index ventricular arrhythmia of unclear cause remained significantly associated with appropriate therapy (HR 0.32 [95% CI 0.12–0.86]; *p* = 0.023) and inappropriate shock (HR 3.72 [95% CI 1.17–11.85]; *p* = 0.026) (Table 5).

### 3.5. Idiopathic VF

For the idiopathic VF group, there was a significant association with appropriate ICD therapy univariably (HR 0.28 [95% CI 0.09–0.90]; *p* = 0.032), but significance was lost after correction for potential confounders (multivariable HR 0.48 [95% CI 0.14–1.65]; *p* = 0.245) (Table S2). A more comprehensive presentation of all secondary analyses can be found in the Supplementary Materials.

## 4. Discussion

In this study, we demonstrate that patients who received an ICD for secondary prevention with an unclear cause of their index ventricular arrhythmia experienced fewer appropriate ICD therapies and more inappropriate shocks than patients with a clear etiology of the index arrhythmia. The rate of first appropriate ICD therapy per 100 person-years was highest in the first two years of follow-up and declined to a lower but more constant rate thereafter. The rate of first inappropriate shock per 100 person-years was constant over time.

Index ventricular arrhythmia of unclear cause was significantly associated with appropriate ICD therapy, with fewer appropriate therapies occurring in this group than in the group with a clear cause of ventricular arrhythmia. In our sensitivity analysis with multiply imputed datasets, this association remained significant. After exclusion of patients with a potential but uncertain etiology, there was still a lower number of appropriate ICD therapies in univariable analysis, but significance was lost in multivariable analysis, potentially due to a loss of statistical power. Previous studies found higher cumulative rates of appropriate therapy in patients with idiopathic VF than reported in our study, with ventricular arrhythmia recurrence rates generally lying between 30% and 43% [17,18]. A meta-analysis conducted by Ozaydin et al. found a recurrence rate of 31% during a follow-up period of 5.3 years [20]. However, one study found a lower rate, with 11% of patients receiving appropriate shocks, although this was during a median follow-up of 2.40 years, considerably shorter than the follow-up time of our study [6,7]. Groeneveld et al. have shown a rate of appropriate ICD therapy of 26% during a median follow-up of 6 years in patients with idiopathic VF. Furthermore, ICD therapies seemed to occur more frequently in patients in whom an alternative diagnosis was found during follow-up than in those for whom this was not the case, with a nearly significant difference between the groups [9]. Another study by William et al., which demonstrated that diagnostic testing is frequently incomplete in patients with unexplained ventricular tachyarrhythmias, found that ICD therapies occurred more frequently in patients with an unexplained ventricular arrhythmia than in those with a clear etiology of ventricular arrhythmia, which is in contrast with our findings [8]. However, it must also be mentioned that William et al. excluded patients older than 60 years of age, who may more often have a clear etiology, whereas in our study the median age was over 60. Furthermore, the rate of ICD therapies in the group of patients with a clear etiology as found by William et al. was relatively low, namely 14.3%, which contrasts with findings from other studies, which generally found higher rates [13,16].

One possible explanation for the lower number of appropriate therapies in patients with a ventricular arrhythmia of unclear cause may be that the absence of a clear substrate, such as that seen in, for instance, patients with a prior myocardial infarction, results in a lower risk of future ventricular arrhythmias and hence a lower likelihood of appropriate therapies. In addition, psychological stress, which may function as a short-lived substrate, may play a role in idiopathic VF [21]. Regional differences in the extensiveness of diagnostic testing, as well as in the prevalence of specific underlying substrates, may, in part, account for differences in the rate of appropriate therapies in this patient population. Furthermore, earlier studies have found the presence of myocardial edema in survivors of SCD to be associated with a low rate of appropriate ICD therapies [22,23]. It may be that patients with myocardial edema constitute a substantial proportion of those with an unclear cause of ventricular arrhythmia, who therefore have a relatively favorable outcome. However, it must also be noted that microstructural abnormalities in the myocardium or Purkinje system may play an important role in a large number of patients with idiopathic VF [24].

A significant association was found between index ventricular arrhythmia of unclear cause and inappropriate ICD shocks, with the risk of inappropriate shocks being higher among patients with an unclear cause of ventricular arrhythmia. This finding is consistent with previous studies, which have shown the rate of inappropriate shocks to be high among patients with idiopathic VF, ranging between 14% and 44% [6,7,9,17,18]. Although no significant association was found for the outcome of inappropriate shocks when taking into consideration only patients with idiopathic VF, the percentage of patients with inappropriate shocks in the idiopathic VF group was nearly double that of those in the non-idiopathic group in our study. This finding could be explained by the fact that patients with an unclear cause of ventricular arrhythmia were generally younger than patients with a clear cause of ventricular arrhythmia. Earlier studies found age to be significantly associated with the occurrence of inappropriate shocks [25,26]. Additionally, considering the fact that inappropriate shocks can be caused by atrial tachyarrhythmias, the fact that fewer patients with an unclear cause of ventricular arrhythmia use antiarrhythmic medication compared with those with a clear cause of ventricular arrhythmia may, in part, account for the observed difference in the rate of inappropriate shocks. Since inappropriate shocks are associated with anxiety and even mortality [25,27], this finding is of clinical importance, since it indicates that patients with an unclear cause of ventricular arrhythmia may be at higher risk of experiencing the disadvantages of an ICD.

Our finding that patients with ventricular arrhythmia of unclear cause have a lower risk of appropriate ICD therapies raises the question of whether the benefits outweigh the risks for these patients, especially considering the fact that inappropriate shocks seemed to occur more frequently in this patient population. The benefit-to-risk ratio of ICD therapy may therefore be less favorable in these patients. Since the rate of first appropriate therapies is highest in the first period after ICD implantation, whereas the rate of first inappropriate shocks is relatively constant over time, it may be that patients with an unclear cause of ventricular arrhythmia who do not experience a first appropriate therapy in the first few years after ICD implantation do not benefit as much from an ICD, so that ICD replacement may not always be in the interest of the patient. In a population of mostly secondary prevention ICD recipients whose device was replaced, the INSURE trial found that a considerable number of patients without prior appropriate device therapy still received appropriate therapies after ICD replacement, even though they had a lower cumulative rate of appropriate therapy than those with prior appropriate therapy [28]. Results from another study conducted with primary prevention patients found that appropriate ICD therapy occurred in 11% and inappropriate therapies occurred in 8% of patients after replacement of the ICD [29]. A similar study may be able to elucidate the balance between risks and benefits of ICD replacement in patients with idiopathic VF. In any case, it is important to explain the possible advantages and disadvantages of ICD replacement to patients, especially since an earlier study demonstrated that a large number of patients with an ICD were not aware that replacement of their device was optional [30].

The strength of our study lies in a relatively long period of follow-up. There were also several limitations, however. First, the sample size of the group with an unclear cause of ventricular arrhythmia was relatively small, although consistent with several previous studies [6]. Missing data may have led to bias, although bias resulting from missing data was reduced by performing a sensitivity analysis with multiply imputed datasets. Although confounding was minimized by correcting for potential confounders in multivariable analyses, there may have been residual confounding.

In conclusion, patients with an unclear cause of ventricular arrhythmia had significantly fewer appropriate ICD therapies than patients with a clear etiology of their ventricular arrhythmia. Further research is required to clarify whether the benefits associated with appropriate therapies outweigh the risks of inappropriate shocks in this patient population.

## Figures and Tables

**Table 1:** Patient characteristics.

Variable	Total Study Population (*n* = 257)	Ventricular Arrhythmia with Clear Cause (*n* = 220)	Ventricular Arrhythmia with Unclear Cause (*n* = 37)	*p*-Value
Age (years)	64.1 (52.9–71.8)	65.2 (56.0–72.0)	52.3 (43.4–63.9)	**<0.001**
Female sex	45 (17.5%)	34 (15.5%)	11 (29.7%)	**0.035**
Presenting ventricular arrhythmia				**0.007**
VF	173 (67.3%)	141 (64.1%)	32 (86.5%)	
Sustained VT	84 (32.7%)	79 (35.9%)	5 (13.5%)	
Type of ICD				**<0.001**
S-ICD	12 (4.7%)	4 (1.8%)	8 (21.6%)	
VVI-ICD	121 (47.1%)	102 (46.4%)	19 (51.4%)	
DDD-ICD	88 (34.2%)	78 (35.5%)	10 (27.0%)	
CRT-D	36 (14.0%)	36 (16.4%)	0 (0.0%)	
BMI (kg/m^2^)	26.5 (24.4–29.4)	26.8 (24.6–29.4)	25.3 (23.9–30.1)	0.216
NYHA class				0.139
I or II	175 (68.1%)	149 (67.7%)	26 (70.3%)	
III or IV	30 (11.7%)	29 (13.2%)	1 (2.7%)	
Medical history				
Myocardial infarction	137 (53.3%)	137 (62.3%)	0 (0.0%)	**<0.001**
DM	48 (18.7%)	43 (19.5%)	5 (13.5%)	0.384
Syncope	24 (9.3%)	20 (9.1%)	4 (10.8%)	0.760
Prior heart surgery	66 (25.7%)	66 (30.0%)	0 (0.0%)	**<0.001**
Atrial fibrillation	86 (33.5%)	77 (35.0%)	9 (24.3%)	0.203
Non-sustained VT	47 (18.3%)	40 (18.2%)	7 (18.9%)	0.915
CAD				**<0.001**
No	93 (36.2%)	59 (26.8%)	34 (91.9%)	
1 coronary artery	39 (15.2%)	36 (16.4%)	3 (8.1%)	
2 coronary arteries	54 (21.0%)	54 (24.5%)	0 (0.0%)	
3 coronary arteries	64 (24.9%)	64 (29.1%)	0 (0.0%)	
Echocardiography				
LVEF (%)	45 (34–53)	41 (33–50)	55 (51–58)	**<0.001**
LGE on CMR				**<0.001**
Yes	80 (31.1%)	73 (33.2%)	7 (18.9%)	
No	53 (20.6%)	27 (12.3%)	26 (70.3%)	
Electrocardiography				
Rhythm				0.406
Sinus rhythm	221 (86.0%)	187 (85.0%)	34 (91.9%)	
Atrial fibrillation	28 (10.9%)	26 (11.8%)	2 (5.4%)	
Atrial flutter	2 (0.8%)	2 (0.9%)	0 (0.0%)	
Pacemaker	3 (1.2%)	2 (0.9%)	1 (2.7%)	
QRS fragmentation				**<0.001**
Yes	90 (35.0%)	77 (35.0%)	13 (35.1%)	
QRS > 120 ms	74 (28.8%)	72 (32.7%)	2 (5.4%)	
Early repolarization	15 (5.8%)	12 (5.5%)	3 (8.1%)	0.463
Laboratory values				
eGFR	77 ± 23	75 ± 23	91 ± 21	**<0.001**
(mL/min/1.73 m^2^)				
Medication at baseline				
ACE-I/ARB	113 (44.0%)	106 (48.2%)	7 (18.9%)	**<0.001**
β-blocker	125 (48.6%)	118 (53.6%)	7 (18.9%)	**<0.001**
Calcium antagonist	43 (16.7%)	39 (17.7%)	4 (10.8%)	0.267
Diuretic	56 (21.8%)	54 (24.5%)	2 (5.4%)	**0.007**
Statin	111 (43.2%)	107 (48.6%)	4 (10.8%)	**<0.001**
MRA	22 (8.6%)	22 (10.0%)	0 (0.0%)	0.052
Class 3	15 (5.8%)	15 (6.8%)	0 (0.0%)	0.137
antiarrhythmic drugs				
Digoxin	9 (3.5%)	9 (4.1%)	0 (0.0%)	0.364

Data are expressed as *n* (%) in case of categorical data, mean ± standard deviation (SD) in case of normally distributed continuous data and median and interquartile range (IQR) in case of continuous data with a skewed distribution. In some cases, numbers may not add up to 100% due to missing data. The presented *p*-values reflect a comparison between patients with ventricular arrhythmia of clear and unclear cause and are presented in bold if *p* < 0.05. ACE-I, angiotensin-converting enzyme inhibitor; ARB, angiotensin receptor blocker; BMI, body mass index; CAD, coronary artery disease; CMR, cardiac magnetic resonance; CRT-D, cardiac resynchronization therapy ICD; DDD-ICD, dual-chamber ICD; DM, diabetes mellitus; eGFR, estimated glomerular filtration rate; ICD, implantable cardioverter-defibrillator; LGE, late gadolinium enhancement; LVEF, left ventricular ejection fraction; MRA, mineralocorticoid receptor antagonist; NYHA, New York Heart Association; S-ICD, subcutaneous ICD; VF, ventricular fibrillation; VT, ventricular tachycardia; VVI-ICD, single-chamber ICD.

**Table 2:** Diagnostic tests performed for patients with ventricular arrhythmia of unclear cause and idiopathic VF.

Diagnostics	Ventricular Arrhythmia of Unclear Cause (*n* = 37)	Idiopathic VF (*n* = 21) *
History taking	37 (100.0%)	21 (100.0%)
Physical examination	37 (100.0%)	21 (100.0%)
Electrocardiography	37 (100.0%)	21 (100.0%)
Laboratory analysis	37 (100.0%)	21 (100.0%)
Toxicology	7 (18.9%)	7 (33.3%)
Echocardiography	37 (100.0%)	21 (100.0%)
Telemetry/Holter	37 (100.0%)	21 (100.0%)
Exercise testing	15 (40.5%)	9 (42.9%)
Cardiac MRI	33 (89.2%)	18 (85.7%)
Ajmaline provocation test	9 (24.3%)	7 (33.3%)
Coronary artery CT angiography	4 (10.8%)	2 (9.5%)
Coronary angiography	34 (91.9%)	19 (90.5%)
Genetic testing	19 (51.4%)	13 (61.9%)

All diagnostic tests stated were performed within 6 months prior to and 3 months after the index event, with the exception of genetic testing and ajmaline provocation test, which could be performed at any time point after the index event. CT, computed tomography; MRI, magnetic resonance imaging; VF, ventricular fibrillation. * The population with idiopathic VF is a subset of the population with a ventricular arrhythmia of unclear cause.

**Table 3:** Number of outcomes during follow-up.

Outcomes	Total Study Population (*n* = 257)	Ventricular Arrhythmia with Clear Cause (*n* = 220)	Ventricular Arrhythmia with Unclear Cause (*n* = 37)	Idiopathic VF (*n* = 21) *
Appropriate ICD therapy	95 (37.0%)	90 (40.9%)	5 (13.5%)	3 (14.3%)
Appropriate shock	72 (28.0%)	67 (30.5%)	5 (13.5%)	3 (14.3%)
Appropriate ATP	78 (30.4%)	77 (35.0%)	1 (2.7%)	1 (4.8%)
All-cause mortality	59 (23.0%)	57 (25.9%)	2 (5.4%)	1 (4.8%)
Cardiac cause of death	14 (5.4%)	14 (6.4%)	0 (0.0%)	0 (0.0%)
Non-cardiac cause of death	14 (5.4%)	13 (5.9%)	1 (2.7%)	0 (0.0%)
Unknown cause of death	31 (12.1%)	30 (13.6%)	1 (2.7%)	1 (4.8%)
Inappropriate ICD therapy	26 (10.1%)	20 (9.1%)	6 (16.2%)	3 (14.3%)
Inappropriate shocks	17 (6.6%)	12 (5.5%)	5 (13.5%)	2 (9.5%)
Complications	20 (7.8%)	15 (6.8%)	5 (13.5%)	3 (14.3%)
Lead failure	12 (4.7%)	7 (3.2%)	5 (13.5%)	3 (14.3%)
Perioperative	8 (3.1%)	7 (3.2%)	1 (2.7%)	0 (0.0%)
complications				
Infection	0 (0.0%)	0 (0.0%)	0 (0.0%)	0 (0.0%)
Other complications	1 (0.4%)	1 (0.5%)	0 (0.0%)	0 (0.0%)

Data are expressed as *n* (%) and reflect the first occurrence of outcomes. ATP, antitachycardia pacing; ICD, implantable cardioverter-defibrillator; VF, ventricular fibrillation. Cause/reason for inappropriate therapy: AF in 18 (69.2%) patients, regular SVT in 7 (26.9%) patients and noise in 1 (3.8%) patient. Cause/reason for inappropriate shock: AF in 13 (76.5%) patients, regular SVT in 3 (17.6%) patients and noise in 1 (5.9%) patient. * The population with idiopathic VF is a subset of the population with a ventricular arrhythmia of unclear cause.

**Table 4:** Cox regression with ventricular arrhythmia of unclear cause as determinant of the outcomes, both univariably and after adjusting for potential confounders.

	Univariable		Adjusted	
Outcome	HR (95% CI)	*p*-Value	HR (95% CI)	*p*-Value
Appropriate ICD therapy	0.26 (0.11–0.65)	0.004	0.37 (0.14–0.99) *	0.048
Appropriate ICD shock	0.37 (0.15–0.93)	0.034	0.65 (0.23–1.86) *	0.422
All-cause mortality	0.19 (0.05–0.79)	0.022	0.69 (0.15–3.23) ^§^	0.640
Inappropriate ICD shock	2.37 (0.83–6.73)	0.105	3.71 (1.17–11.80) °	0.026

CI, confidence interval; HR, hazard ratio; ICD, implantable cardioverter-defibrillator. * Adjusted for age, sex, BMI, index arrhythmia, history of atrial fibrillation, prior syncope, history of non-sustained VT, history of myocardial infarction, QRS fragmentation, eGFR and left ventricular ejection fraction. *n* = 227 (88.3%). ^§^ Adjusted for age, sex, index arrhythmia, diabetes, history of myocardial infarction, history of heart surgery and eGFR. *n* = 253 (98.4%). ° Adjusted for age, sex and rhythm on ECG. *n* = 254 (98.8%).

**Table 5:** Cox regression with ventricular arrhythmia of unclear cause as determinant of the outcomes after adjusting for potential confounders, using multiple imputation.

	Adjusted	
Outcome	HR (95% CI)	*p*-Value
Appropriate ICD therapy	0.32 (0.12–0.86) *	0.023
Appropriate ICD shock	0.54 (0.19–1.50) *	0.235
All-cause mortality	0.66 (0.14–3.03) ^§^	0.592
Inappropriate ICD shock	3.72 (1.17–11.85) °	0.026

CI, confidence interval; HR, hazard ratio; ICD, implantable cardioverter-defibrillator. * Adjusted for age, sex, BMI, index arrhythmia, history of atrial fibrillation, prior syncope, history of non-sustained VT, history of myocardial infarction, QRS fragmentation, eGFR and left ventricular ejection fraction. ^§^ Adjusted for age, sex, index arrhythmia, diabetes, history of myocardial infarction, history of heart surgery and eGFR. ° Adjusted for age, sex and rhythm on ECG.

## Data Availability

The data presented in this study are available on request from the corresponding author. The data are not publicly available due to ongoing analyses.

## Supplementary Materials

The following supporting information can be downloaded at: https://www.mdpi.com/article/10.3390/jcm12134479/s1, Figure S1: Kaplan–Meier curves displaying cumulative event-free survival for the idiopathic VF and non-idiopathic groups with appropriate ICD therapy, appropriate ICD shock, all-cause mortality and inappropriate ICD shock as outcomes; Table S1: Patient characteristics; Table S2: Cox regression with idiopathic VF as determinant of the outcomes, both univariably and after adjusting for potential confounders; Table S3: Cox regression with idiopathic VF as determinant of the outcomes after adjusting for potential confounders, using multiple imputation.
