# Supplementary material for: Appropriate Implantable Cardioverter-Defibrillator Therapy in Patients with Ventricular Arrhythmia of Unclear Cause in Secondary Prevention of Sudden Cardiac Death
Source: J Clin Med. 2023 Jul 4;12(13):4479. doi: 10.3390/jcm12134479 (PMC10342351; doi:10.3390/jcm12134479)
Supplement: Supplementary file 1 [file jcm-12-04479-s001.zip › jcm-2472850-supplementary.pdf]

Table S1. Patient characteristics.				
**Variable**	**Total study population (*n* = 257)**	**Ventricular arrhythmia with clear cause (*n* = 220)**	**Ventricular arrhythmia with unclear cause (*n* = 37)**	***P*-value**
Age (years)	64.1 (52.9 – 71.8)	65.2 (56.0 – 72.0)	52.3 (43.4 – 63.9)	**<0.001**
Female sex	45 (17.5%)	34 (15.5%)	11 (29.7%)	**0.035**
Presenting ventricular arrhythmia				**0.007**
VF	173 (67.3%)	141 (64.1%)	32 (86.5%)	
Sustained VT	84 (32.7%)	79 (35.9%)	5 (13.5%)	
OHCA	182 (70.8%)	149 (67.7%)	33 (89.2%)	**0.008**
Type of ICD				**<0.001**
S-ICD	12 (4.7%)	4 (1.8%)	8 (21.6%)	
VVI-ICD	121 (47.1%)	102 (46.4%)	19 (51.4%)	
DDD-ICD	88 (34.2%)	78 (35.5%)	10 (27.0%)	
CRT-D	36 (14.0%)	36 (16.4%)	0 (0.0%)	
BMI (kg/m^2)	26.5 (24.4 – 29.4)	26.8 (24.6 – 29.4)	25.3 (23.9 – 30.1)	0.216
NYHA class				0.139
I or II	175 (68.1%)	149 (67.7%)	26 (70.3%)	
III or IV	30 (11.7%)	29 (13.2%)	1 (2.7%)	
Medical history				
Hypertension	124 (48.2%)	108 (49.1%)	16 (43.2%)	0.510
Dyslipidemia	96 (37.4%)	90 (40.9%)	6 (16.2%)	**0.004**
Myocardial infarction	137 (53.3%)	137 (62.3%)	0 (0.0%)	**<0.001**
DM	48 (18.7%)	43 (19.5%)	5 (13.5%)	0.384
Family history SCD	24 (9.3%)	22 (10.0%)	2 (5.4%)	0.545
Syncope	24 (9.3%)	20 (9.1%)	4 (10.8%)	0.760
Prior heart surgery	66 (25.7%)	66 (30.0%)	0 (0.0%)	**<0.001**
Atrial fibrillation	86 (33.5%)	77 (35.0%)	9 (24.3%)	0.203
Non-sustained VT	47 (18.3%)	40 (18.2%)	7 (18.9%)	0.915
CAD				**<0.001**
No	93 (36.2%)	59 (26.8%)	34 (91.9%)	
1 coronary artery	39 (15.2%)	36 (16.4%)	3 (8.1%)	
2 coronary arteries	54 (21.0%)	54 (24.5%)	0 (0.0%)	
3 coronary arteries	64 (24.9%)	64 (29.1%)	0 (0.0%)	
Echocardiography				
LVEF (%)	45 (34 – 53)	41 (33 – 50)	55 (51 – 58)	**<0.001**
LAVI (mL/m^2)	35 (29 – 45)	37 (30 – 45)	31 (25 – 38)	**0.015**
Maximum myocardial wall thickness (mm)	10.9 ± 2.1	10.9 ± 2.1	10.8 ± 2.2	0.883
LVEDD (mm)	55.1 ± 8.5	55.8 ± 8.6	50.9 ± 6.6	**0.002**
LV mass (g)	213 ± 64	218 ± 64	185 ± 57	**0.018**
RVF				0.202
Poor	3 (1.2%)	3 (1.4%)	0 (0.0%)	
Mediocre	6 (2.3%)	6 (2.7%)	0 (0.0%)	
Reasonable	36 (14.0%)	34 (15.5%)	2 (5.4%)	
Good	195 (75.9%)	160 (72.7%)	35 (94.6%)	
Mitral insufficiency				**0.002**
None	153 (59.5%)	121 (55.0%)	32 (86.5%)	
Mild (grade 1)	61 (23.7%)	58 (26.4%)	3 (8.1%)	
Moderate (grade 2)	20 (7.8%)	20 (9.1%)	0 (0.0%)	
Severe (grade 3)	5 (1.9%)	4 (1.8%)	1 (2.7%)	
LGE on CMR				**<0.001**
Yes	80 (31.1%)	73 (33.2%)	7 (18.9%)	
No	53 (20.6%)	27 (12.3%)	26 (70.3%)	
Electrocardiography				
Rhythm				0.406
Sinus rhythm	221 (86.0%)	187 (85.0%)	34 (91.9%)	

Atrial fibrillation	28 (10.9%)	26 (11.8%)	2 (5.4%)	
Atrial flutter	2 (0.8%)	2 (0.9%)	0 (0.0%)	
Pacemaker	3 (1.2%)	2 (0.9%)	1 (2.7%)	
Ventricular heart rate (bpm)	67 ± 14	67 ± 14	69 ± 12	0.370
PR interval (ms)	168 (152 – 188)	168 (152 – 190)	164 (148 – 186)	0.338
QRS duration (ms)	108 (98 – 128)	111 (98 – 133)	96 (88 – 106)	**<0.001**
QRS axis (degrees)	31 (-13 – 65)	30 (-18 – 64)	37 (-1 – 74)	0.072
QT interval (ms)	434 ± 51	441 ± 52	399 ± 33	**<0.001**
QRS fragmentation				**<0.001**
Yes	90 (35.0%)	77 (35.0%)	13 (35.1%)	
QRS > 120ms	74 (28.8%)	72 (32.7%)	2 (5.4%)	
QRS morphology				0.062
Normal	140 (54.5%)	111 (50.5%)	29 (78.4%)	
LBBB	25 (9.7%)	24 (10.9%)	1 (2.7%)	
LAFB	38 (14.8%)	34 (15.5%)	4 (10.8%)	
LPFB	4 (1.6%)	3 (1.4%)	1 (2.7%)	
RBBB	14 (5.4%)	13 (5.9%)	1 (2.7%)	
RBBB + LAFB	10 (3.9%)	10 (4.5%)	0 (0.0%)	
RBBB + LPFB	3 (1.2%)	3 (1.4%)	0 (0.0%)	
IVCD	18 (7.0%)	18 (8.2%)	0 (0.0%)	
Pacing	3 (1.2%)	2 (0.9%)	1 (2.7%)	
Early repolarization	15 (5.8%)	12 (5.5%)	3 (8.1%)	0.463
Laboratory values				
Hb (mmol/L)	8.1 ± 1.2	8.1 ± 1.2	8.3 ± 1.1	0.480
Na^+ (mmol/L)	140 (138 – 141)	140 (138 – 141)	140 (139 – 141)	0.933
K^+ (mmol/L)	4.2 ± 0.4	4.2 ± 0.4	4.2 ± 0.4	0.460
Creatinine (μmol/L)	86 (75 – 100)	88 (77 – 104)	75 (64 – 87)	**<0.001**
eGFR (mL/min/1.73m^2)	77 ± 23	75 ± 23	91 ± 21	**<0.001**
Urea (mmol/L)	6.2 (4.9 – 7.7)	6.3 (4.9 – 8.1)	5.3 (4.6 – 6.8)	**0.008**
NT-proBNP (ng/L)	656 (226 – 1,625)	891 (295 – 1,831)	155 (46 – 330)	**<0.001**
Medication at baseline				
ACE-I/ARB	113 (44.0%)	106 (48.2%)	7 (18.9%)	**<0.001**
β-blocker	125 (48.6%)	118 (53.6%)	7 (18.9%)	**<0.001**
Calcium antagonist	43 (16.7%)	39 (17.7%)	4 (10.8%)	0.267
Diuretic	56 (21.8%)	54 (24.5%)	2 (5.4%)	**0.007**
Statin	111 (43.2%)	107 (48.6%)	4 (10.8%)	**<0.001**
MRA	22 (8.6%)	22 (10.0%)	0 (0.0%)	0.052
Class 1 antiarrhythmic drugs	1 (0.4%)	1 (0.5%)	0 (0.0%)	1.000
Class 3 antiarrhythmic drugs	15 (5.8%)	15 (6.8%)	0 (0.0%)	0.137
Digoxin	9 (3.5%)	9 (4.1%)	0 (0.0%)	0.364

Data are expressed as *n* (%) in case of categorical data, mean ± standard deviation (SD) in case of normally distributed continuous data and median and interquartile range (IQR) in case of continuous data with a skewed distribution. In some cases, numbers may not add up to 100% due to missing data. The presented *P*-values reflect a comparison between patients with ventricular arrhythmia of clear and unclear cause and are presented in bold if $P < 0.05$. ACE-I, angiotensin-converting enzyme inhibitor; ARB, angiotensin receptor blocker; BMI, body mass index; CAD, coronary artery disease; CMR, cardiac magnetic resonance; CRT-D, cardiac resynchronization therapy ICD; DDD-ICD, dual-chamber ICD; DM, diabetes mellitus; eGFR, estimated glomerular filtration rate; Hb, hemoglobin; ICD, implantable cardioverter-defibrillator; IVCD, intraventricular conduction delay; LAFB, left anterior fascicular block; LAVI, left atrial volume index; LBBB, left bundle branch block; LGE, late gadolinium enhancement; LPFB, left posterior fascicular block; LV, left ventricle; LVEDD, left ventricular end-diastolic diameter; LVEF, left ventricular ejection fraction; MRA, mineralocorticoid receptor antagonist; NT-proBNP, N-terminal pro-brain natriuretic peptide; NYHA, New York Heart Association; OHCA, out-of-hospital cardiac arrest; RBBB, right bundle branch block; RVF, right ventricular function; SCD, sudden cardiac death; S-ICD, subcutaneous ICD; VF, ventricular fibrillation; VT, ventricular tachycardia; VVI-ICD, single-chamber ICD.

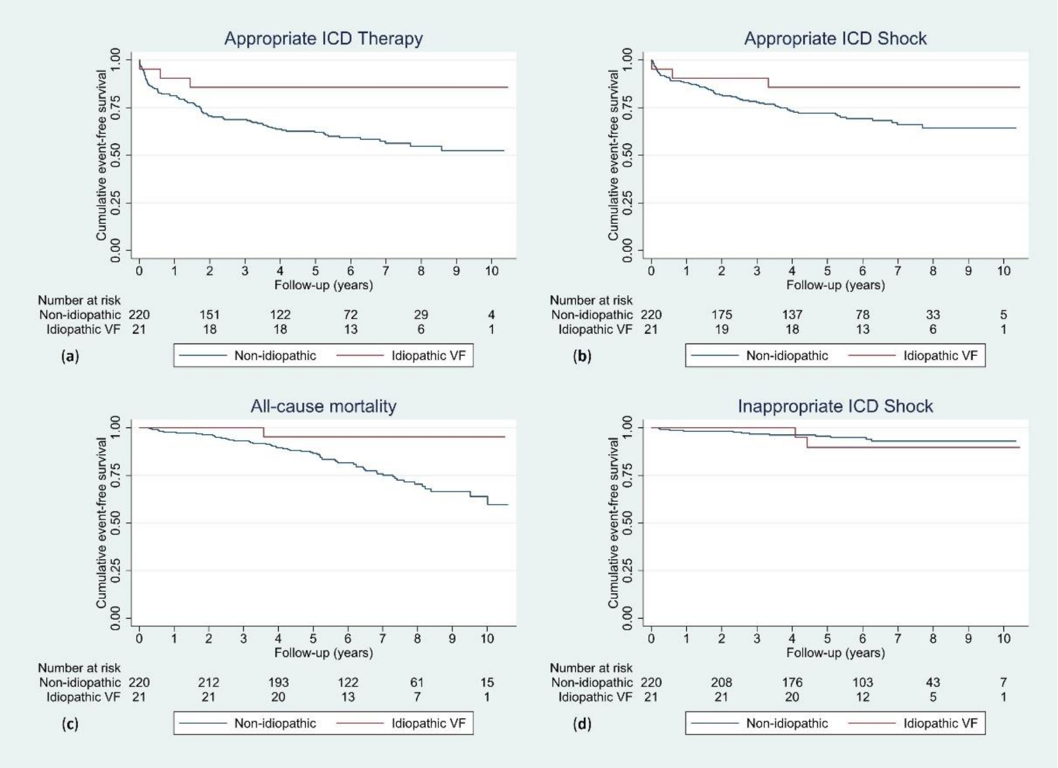

Figure S1. Kaplan-Meier curves displaying cumulative event-free survival for the idiopathic VF and non-idiopathic groups with appropriate ICD therapy (log-rank test *P* = 0.022) (**a**), appropriate ICD shock (log-rank test *P* = 0.109) (**b**), all-cause mortality (log-rank test *P* = 0.045) (**c**) and inappropriate ICD shock (log-rank test *P* = 0.529) (**d**) as outcomes. ICD, implantable cardioverter-defibrillator; VF, ventricular fibrillation.

Table S2. Cox regression with idiopathic VF as determinant of the outcomes, both univariably and after adjusting for potential confounders.

	**Univariable**		**Adjusted**	
**Outcome**	**HR (95% CI)**	***P*-value**	**HR (95% CI)**	***P*-value**
Appropriate ICD therapy	0.28 (0.09 – 0.90)	0.032	0.48 (0.14 – 1.65)*	0.245
Appropriate ICD shock	0.40 (0.13 – 1.28)	0.122	0.75 (0.21 – 2.72)*	0.659
All-cause mortality	0.17 (0.02 – 1.22)	0.078	0.70 (0.09 – 5.65)§	0.741
Inappropriate ICD shock	1.61 (0.36 – 7.20)	0.532	2.28 (0.44 – 11.80)º	0.328

CI, confidence interval; HR, hazard ratio; ICD, implantable cardioverter-defibrillator; VF, ventricular fibrillation.
* Adjusted for age, sex, BMI, index arrhythmia, history of atrial fibrillation, prior syncope, history of non-sustained VT, history of myocardial infarction, QRS fragmentation, eGFR and left ventricular ejection fraction.
§ Adjusted for age, sex, index arrhythmia, diabetes, history of myocardial infarction, history of heart surgery and eGFR.
º Adjusted for age, sex and rhythm on ECG.

**Table S3. Cox regression with idiopathic VF as determinant of the outcomes after adjusting for potential confounders, using multiple imputation.**		
	**Adjusted**	
**Outcome**	**HR (95% CI)**	***P*-value**
Appropriate ICD therapy	0.42 (0.12 – 1.42)*	0.162
Appropriate ICD shock	0.62 (0.18 – 2.20)*	0.460
All-cause mortality	0.69 (0.09 – 5.47)§	0.723
Inappropriate ICD shock	2.27 (0.44 – 11.80)º	0.328

CI, confidence interval; HR, hazard ratio; ICD, implantable cardioverter-defibrillator; VF, ventricular fibrillation.

* Adjusted for age, sex, BMI, index arrhythmia, history of atrial fibrillation, prior syncope, history of non-sustained VT, history of myocardial infarction, QRS fragmentation, eGFR and left ventricular ejection fraction.

§ Adjusted for age, sex, index arrhythmia, diabetes, history of myocardial infarction, history of heart surgery and eGFR.

º Adjusted for age, sex and rhythm on ECG.
